# Microscope-based augmented reality with diffusion tensor imaging and fluorescein in insular glioma resection

**DOI:** 10.3171/2021.10.FOCVID2157

**Published:** 2022-01-01

**Authors:** Sabino Luzzi, Alice Giotta Lucifero

**Affiliations:** 1 Neurosurgery Unit, Department of Clinical-Surgical, Diagnostic and Pediatric Sciences, University of Pavia; and; 2 Neurosurgery Unit, Department of Surgical Sciences, Fondazione IRCCS Policlinico San Matteo, Pavia, Italy

**Keywords:** augmented reality, diffusion tensor imaging, fluorescein, insular glioma, neuronavigation

## Abstract

Maximal safe resection is the goal of insular glioma surgery. The combination of intraoperative augmented reality (AR) diffusion tensor imaging (DTI) fiber tracking with fluorescein dye (F) helps achieve this goal throughout a microscope-based visualization of the tumor and white matter fiber tracts.

The aim of the present video article was to show the technical key aspects of DTI-F microscope-based AR-assisted surgery during the gross-total resection of an insular Berger-Sanai type I+IV high-grade glioma in a 63-year-old patient, performed through a pterional transsylvian approach.

The video can be found here: https://stream.cadmore.media/r10.3171/2021.10.FOCVID2157

## Transcript

### 0:21

This video article aims to show the technical key aspects of the microscope-based augmented reality (AR) with diffusion tensor imaging (DTI) and fluorescein in insular glioma resection.

### 0:31

A 63-year-old female patient suffered from a progressive decline in learning and memory, and new-onset epilepsy. The neurological exam revealed a neurocognitive and visuospatial impairment. Past medical history was unremarkable.

### 0:45

MRI revealed a right insular Berger-Sanai type I+IV high-grade glioma.

### 0:51

The tumor arose at the level of the extreme capsule, and it involved the short and long gyri.

### 0:57

The claustrum and the lentiform nucleus were pushed medially, and the medial part of the lesion was in contact with the putamen.

### 1:07

In their progressive growth, insular gliomas tend to encase the lenticulostriate arteries, this aspect being the major cause of morbidity of insular glioma surgery.^1–9^

### 1:18

3D-rendered MRI showed in more detail the anatomical relationships between the tumor, circular sulcus of the insula, and putamen.

### 1:28

Preoperative DTI fiber tracking showed the spatial distribution of the corticospinal tract, occipitofrontal fasciculus, posterior thalamic peduncle, and optic radiation compared to the tumor. It also allowed for the selection of the surgical approach.

### 1:44

A pterional transsylvian approach was selected.

### 1:49

Noteworthy, transsylvian route allows for multiple working corridors.

### 1:54

The different vascular supply of the basal ganglia and insular cortex from the lenticulostriate arteries and MCA, respectively, is to be taken into account during the planning of surgery.

### 2:04

Total intravenous anesthesia target-controlled infusion was used to allow the neuromonitoring. Cortical subcortical mapping was performed with transcranial electric stimulation motor evoked potentials, while subcortical mapping was carried out with the high-frequency bipolar stimulation technique.

### 2:23

Asleep craniotomy was performed. A one-layer submuscular pterional approach was carried out with the full exposure of the lateral part of the sylvian fissure.

### 2:34

Brain mapping is recommended since DTI provides only anatomical information but no data about the function.

### 2:42

The first step of surgery consisted of the opening of the carotid cistern, Liliequist membrane, chiasmatic cistern, along with the fenestration of the lamina terminalis.

### 2:56

Meticulous sharp dissection of the arachnoid membranes is of utmost importance in avoiding any damage to the neurovascular structures of the basal cisterns.

### 3:12

It is also essential to keep clean the surgical field.

### 3:19

A wide opening of the basal cisterns, Liliequist membrane included, allows for CSF withdrawal. This is the key point to avoid the use of fixed brain retraction in the transsylvian approach.

### 3:33

The dynamic retraction by the surgical instruments is less traumatizing and more effective in allowing for the progressive recognition and exposure of the anatomical structures.

### 3:43

The visualization of the lenticulostriate arteries is crucial since they mark the most medial limit of surgery.

### 3:51

Microscope-based AR fiber tractography provided for virtual view of the anatomical relationships between the lateral lenticulostriate arteries and white matter fiber tracts.

### 4:02

The splitting of the lateral and anterior insular compartment of the sylvian fissure and opening of the sylvian cistern allowed to expose the M1 segment of the middle cerebral artery (MCA), MCA bifurcation, superior and inferior limiting sulcus of the insula, limen insula, and entire insular cortex.

### 4:29

In this case, the middle trunk of the MCA originated from the superior trunk.

### 4:38

The insular cortex showed a fluorescein enhancement.

### 4:48

The cortical branches of the MCA were coagulated and the tumor was resected in piecemeal fashion. The use of fluorescein filter aided the recognition of the tumor boundaries.

### 5:02

Care must be taken during tumor removal in avoiding mechanical vasospasm of the MCA or its branches caused by an excessive retraction of the vessels.

### 5:17

The use of the cavitron ultrasonic aspirator facilitated the tumor resection.

### 5:22

DTI allowed for a constant awareness of the spatial location of the corticospinal tract, fronto-occipital fasciculus, and optic radiation.

### 5:30

The accuracy of the AR fiber tractography was checked at a cortical and subcortical level, having the motor strip as a landmark. At the cortical level, the matching between the virtual corticospinal tract and the opercular part of the motor area was tested. At the subcortical level, the matching was verified through the subcortical mapping.

### 5:51

DTI navigation was extremely useful during the resection of the deepest part of the lesion, close to the putamen.

### 5:58

At this step, the lack of fluorescence confirmed the gross-total resection of the tumor.

### 6:06

At the end of surgery, the check of the blood flow ruled out spasm of the MCA and its branches.

### 6:17

ICG videoangiography showed the patency of the MCA.

### 6:30

A gross-total resection of the glioma was achieved, as confirmed by the postoperative MRI. Postoperative DTI documented the preserved structural anatomical connectivity of the corticospinal tract, IFOF, and optic radiation. It was basically as an anatomical confirmation of a functional check which has been already obtained intraoperatively by means of the cortical-subcortical mapping of the motor pathway.

### 6:56

The patient was discharged without deficits on the 7th postoperative day. The relatively longer perioperative follow-up was justified by the known risk of delayed ischemic complications. A urinary tract infection occurred at the 3rd postoperative day, required the administration of intravenous antibiotic therapy. Pathology revealed an anaplastic astrocytoma. Standard Stupp protocol was employed and no recurrence occurred at the 21st-month follow-up.

### 7:25

In conclusion, DTI-fluorescein microscope-based AR resulted safe and effective in maximizing the extent of resection of the reported insular high-grade glioma. Its association with brain mapping is recommended. Further theoretical advantages may also come from its use during awake surgery.

## Acknowledgments

We thank Eng. Giorgia Di Giusto for the technical support.

## Disclosures

The authors report no conflict of interest concerning the materials or methods used in this study or the findings specified in this publication.

## Author Contributions

Primary surgeon: Luzzi. Assistant surgeon: Giotta Lucifero. Editing and drafting the video and abstract: both authors. Critically revising the work: Luzzi. Reviewed submitted version of the work: Luzzi. Approved the final version of the work on behalf of both authors: Luzzi. Supervision: Luzzi.
